# Henipavirus in Northern Short-Tailed Shrew, Alabama, USA

**DOI:** 10.3201/eid3102.241155

**Published:** 2025-02

**Authors:** Rhys H. Parry, KayLene Y.H. Yamada, Wendy R. Hood, Yang Zhao, Jinlong Y. Lu, Andrei Seluanov, Vera Gorbunova, Naphak Modhiran, Daniel Watterson, Ariel Isaacs

**Affiliations:** University of Queensland School of Chemistry and Molecular Biosciences, Brisbane, Queensland, Australia (R.H. Parry, N. Modhiran, D. Watterson, A. Isaacs); Auburn University, Auburn, Alabama, USA (K.Y.H. Yamada, W.R. Hood); University of Rochester, Rochester, New York, USA (Y. Zhao, J.Y. Lu, A. Seluanov, V. Gorbunova)

**Keywords:** Henipavirus, northern short-tailed shrew, *Blarina brevicauda*, Alabama, viruses, zoonoses, United States

## Abstract

RNA metagenomic analysis of tissues from 4 wild-caught northern short-tailed shrews in Alabama, USA, revealed a novel henipavirus (family Paramyxoviridae). Phylogenetic analysis supported the placement of the virus within the shrew henipavirus clade, related to human-infecting shrewborne henipaviruses. Our study results highlight the presence of henipavirus infections in North America.

Henipaviruses (family Paramyxoviridae) are zoonotic, negative-sense RNA viruses harbored primarily by bats. Henipaviruses can cross species barriers, infecting various mammals, including humans; they often cause severe respiratory illness and encephalitis and are associated with high case fatality rates (*1*). The 2 most notable henipaviruses are Hendra virus and Nipah virus. Hendra virus, first identified in Australia, has caused outbreaks with mortality rates up to 70% (*1*). Nipah virus has been linked with numerous outbreaks in Southeast Asia, particularly in Malaysia and Bangladesh, with case-fatality rates estimated at 40%–75% (*1*), depending on surveillance and clinical management.

In 2018, researchers identified a novel henipavirus, Langya virus (LayV), in patients from China’s Shandong and Henan Provinces (*2*). A total of 35 persons were infected with LayV, displaying such symptoms as fever, fatigue, and cough and, in some cases, impaired liver or kidney function. No fatalities have been reported thus far. Most investigators believe the primary reservoir host for LayV to be shrews, but the virus has also been detected in goats and dogs, indicating a wide potential host range.

In 2021, researchers conducting a mammalian longevity study captured 4 northern short-tailed shrews (*Blarina brevicauda*; order: Eulipotyphla, family: Soricidae) in the wild at Camp Hill, Auburn, Alabama, USA (latitude 32.82, longitude −85.65) (*3*). The collection process adhered to protocols approved by the Institutional Animal Care and Use Committee at Auburn University (PRN 2021-3848). Technicians dissected and froze skin, heart, kidney, liver, and brain samples for subsequent analysis. We pulverized the frozen tissues under liquid nitrogen and subjected them to RNA extraction using TRIzol Reagent (Thermo Fisher Scientific, https://www.thermofisher.com). We then treated the RNA with deoxyribonuclease, purified the treated sample, and sequenced the RNA by using the TruSeq Stranded Total RNA RiboZero Gold kit (Illumina, https://www.illumina.com) on an Illumina HiSeq 4000 platform at the New York University Genome Technology Center (New York, NY, USA). We assembled the generated sequence data by using MEGAHIT v1.2.9 software (https://github.com/voutcn/megahit) and subjected the data to virus discovery analysis with BLASTx (https://blast.ncbi.nlm.nih.gov/Blast.cgi?LINK_LOC=blasthome&PAGE_TYPE=BlastSearch&PROGRAM=blastx).

We assembled a single 16,681–16697nt contig from all virus genomes (220, 221, 217, 218; Genbank accesson nos. PQ140948–51) containing conserved *Henipavirus* genome order N-C/P-M-F-G-L, along with a novel open reading frame between M-F, which had been predicted in LayV and other shrew henipaviruses (Figure 1). We noted the largest predicted protein sequence from this contig to be most closely related to the RNA-dependent RNA polymerase of Ninorex virus (Genbank accesson nos. WJL29506.1; identity 74.58%, E-value 0), identified from Eurasian pygmy shrew (*Sorex minutus*) kidney samples in Belgium (*4*) and *Chodsigoa hypsibia* henipavirus from the De Winton’s shrew in China (*5*). Given the divergence from the closest relative, host species, and geographic location, we named the putative virus Camp Hill virus (CHV). CHV reads across all shrews were positive only in kidney tissues, suggesting renal tropism (Appendix Figure 1).

**Figure 1:**
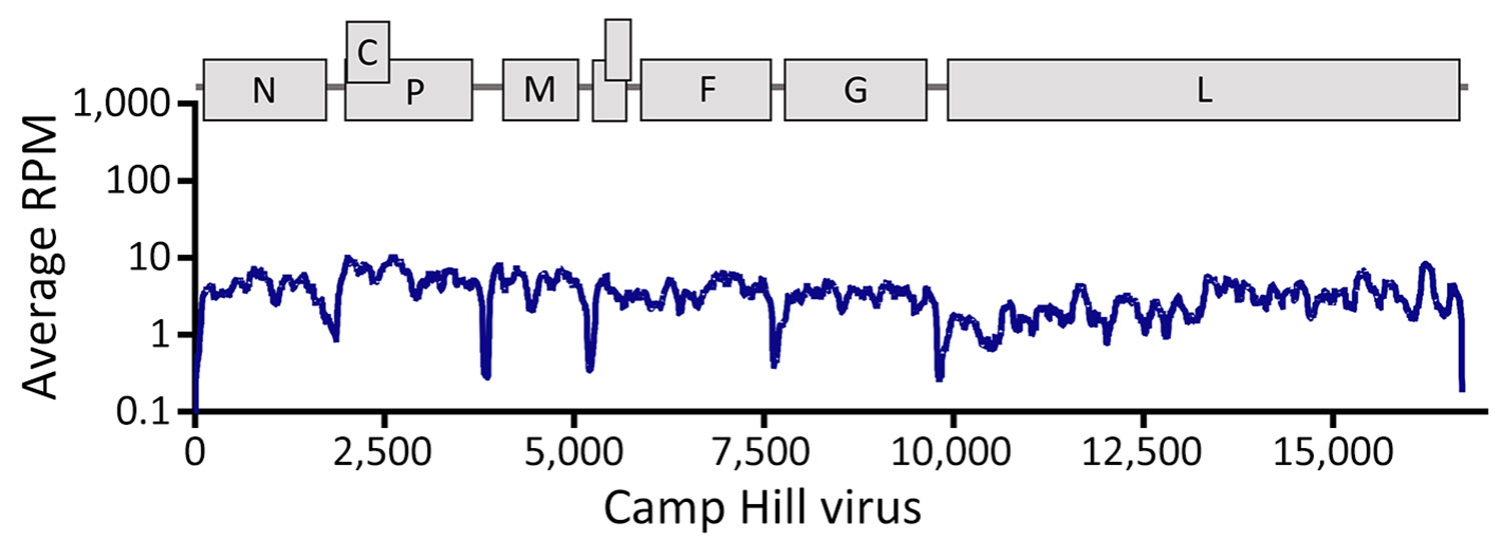
Genome organization and coverage of Camp Hill virus identified in *Blarina brevicauda* shrews from Alabama, USA. Coverage shows subgenomic expression (in RPM) of henipavirus genes (shown across top). RPM, reads per million.

For the phylogenetic analysis of CHV, we aligned the deduced CHV-221 RNA-dependent RNA polymerase protein with 19 other henipaviruses using the MAFFT-G-INS-1 multiple sequence alignment program (https://github.com/GSLBiotech/mafft). We trimmed ambiguous alignments with Gblocks 0.91b (http://phylogeny.lirmm.fr/phylo_cgi/one_task.cgi?task_type=gblocks). A maximum-likelihood phylogeny generated using IQ-TREE v2.1.3 (http://www.iqtree.org) on the final alignment indicated that CHV grouped within a well-supported shrew clade with other shrew henipaviruses from Eurasia (Figure 2) and supported the delineation of CHV as a separate henipavirus on the basis of the species demarcation for the *Henipavirus* genus (International Committee on Taxonomy of Viruses, https://ictv.global/report/chapter/paramyxoviridae/paramyxoviridae/henipavirus).

**Figure 2:**
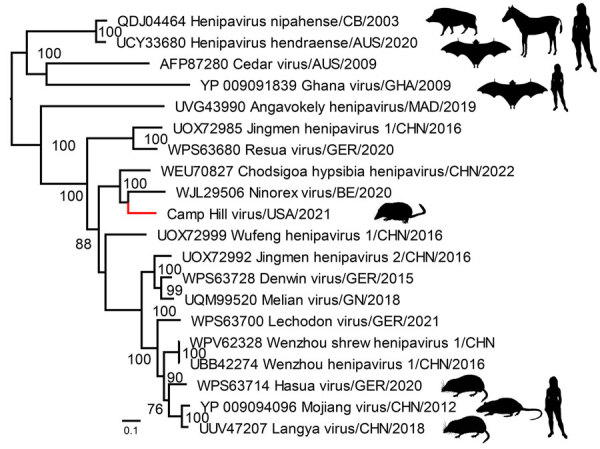
Phylogenetic placement of Camp Hill virus (red) within the *Henipavirus* genus identified in *Blarina brevicauda* shrews from Alabama, USA. GenBank accession numbers are provided for reference sequences. Maximum-likelihood phylogeny is midpoint rooted. Scale bar indicates amino acid substitutions per position.

The discovery of a novel henipavirus in *B. brevicauda* shrews highlights the potential of this shrew species as a zoonotic reservoir, capable of harboring multiple viruses that pose a risk to humans. Of note, the *B. brevicauda* shrew is a known host of Camp Ripley virus (genus *Orthohantavirus*) (*6*,*7*), a viral genus associated with severe human disease. Camp Ripley virus was abundant in tissues from all the individual shrews analyzed, suggesting mixed co-infections of hantaviruses and henipaviruses in the shrews we studied. In addition, a prior report has implicated *B. brevicauda* shrews as reservoir for Powassan virus (genus *Orthoflavivirus*) (*8*), capable of causing life-threatening encephalitis. 

The northern short-tailed shrew is widely distributed across central and eastern North America, from southern Saskatchewan to the Atlantic provinces of Canada and south to northern Arkansas and Georgia in the United States (Appendix Figure 2). Despite their solitary nature, short-tailed shrews are territorial and highly active, commonly found in rural and urban areas near livestock, agricultural settings, and human populations. Although the shrews have large home ranges that sometimes overlap with human activity, they typically inhabit woodland areas with >50% herbaceous cover (*9*), making direct encounters with humans uncommon.

Given the high case-fatality rates associated with henipaviruses, detection of CHV in North America raises concerns about past and potential future spillover events. Further investigation is needed into the potential for human infection and strategies for mitigating transmission. Our findings help elucidate the prevalence and geographic distribution of CHV in *B. brevicauda* shrews. The exact transmission mechanisms of shrew henipaviruses remain unclear, but direct contact with infected animals or their excreta poses a risk to humans.

Appendix Additional information for henipavirus in the northern short-tailed shrew, Alabama, USA
