# Supplementary material for: Henipavirus in Northern Short-Tailed Shrew, Alabama, USA
Source: Emerg Infect Dis. 2025 Feb;31(2):392–4. doi: 10.3201/eid3102.241155 (PMC11845136; doi:10.3201/eid3102.241155)
Supplement: Appendix — Additional information for henipavirus in the northern short-tailed shrew, Alabama, USA [file 24-1155-Techapp-s1.pdf]

Article DOI: http://doi.org/10.3201/eid3102.241155

EID cannot ensure accessibility for supplementary materials supplied by authors. Readers who have difficulty accessing supplementary content should contact the authors for assistance.

Henipavirus in Northern Short-Tailed Shrew, Alabama, USA

Appendix

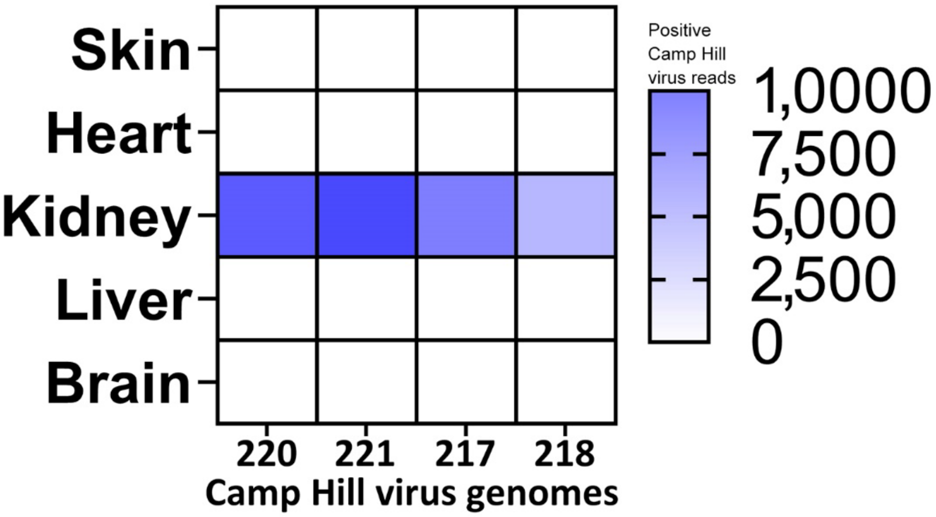

Appendix Figure 1. Tissue tropism of Camp Hill virus across various tissues from *Blarina brevicauda* (genomes 220, 221, 217, 218; Genbank ID PQ140948-PQ140951). Abundance data was derived from trimmed RNA-seq reads mapped to the Camp Hill virus genome using Bowtie2 (https://bowtie-bio.sourceforge.net/bowtie2), illustrating viral distribution across different tissue types.

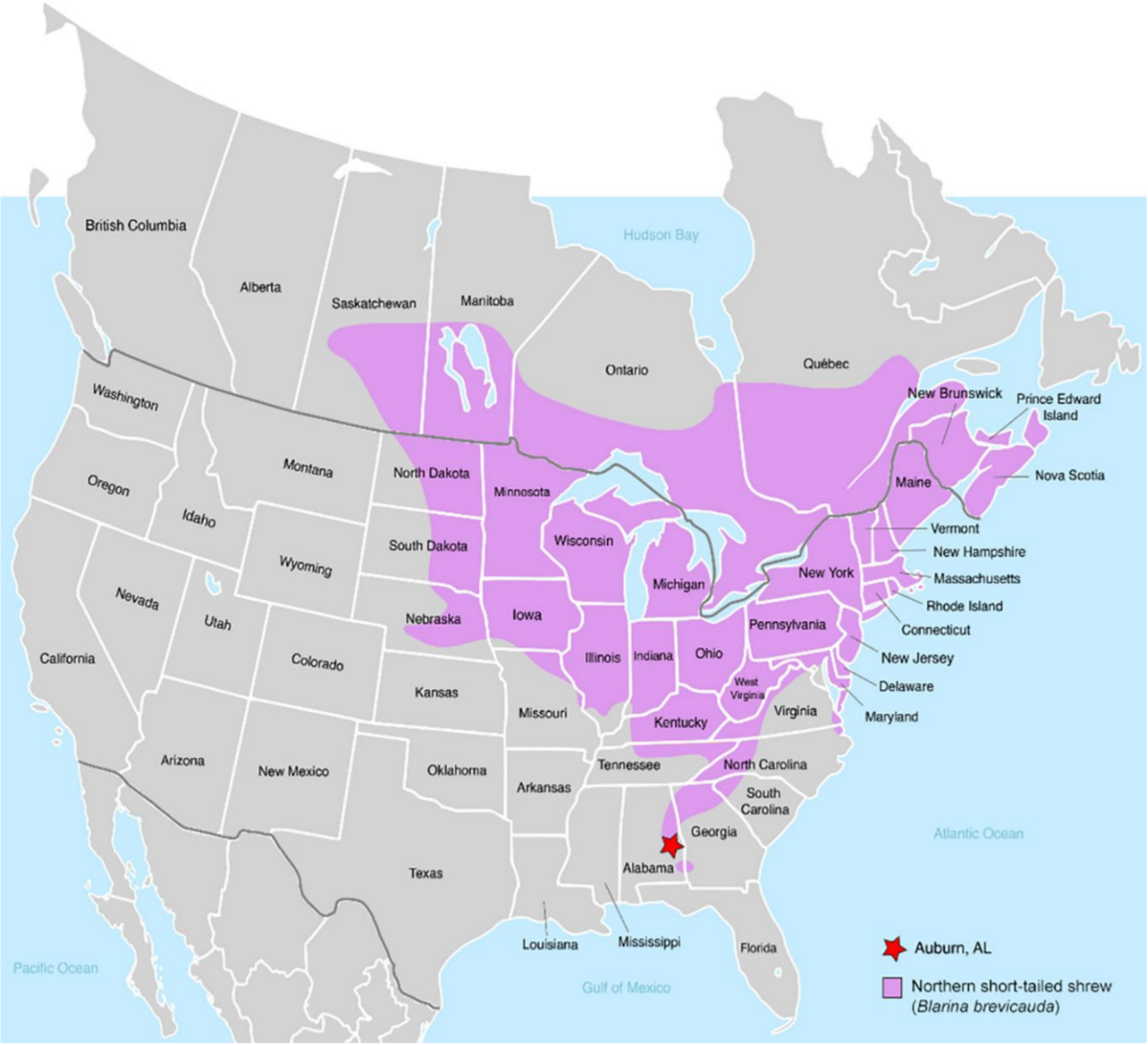

Appendix Figure 2. *Blarina brevicauda* incidence data in North America and Camp Hill virus-positive samples from Auburn, Alabama, USA. Incidence data adapted from *Histoire naturelle des mammifères du Canada, 2016* (Naughton et al, 2016; https://www.editionsmichelquintin.ca/produit/1234-histoire-naturelle-des-mammiferes-du-canada.html).
